# Bacterial whole genome-based phylogeny: construction of a new benchmarking dataset and assessment of some existing methods

**DOI:** 10.1186/s12864-016-3407-6

**Published:** 2017-01-05

**Authors:** Johanne Ahrenfeldt, Carina Skaarup, Henrik Hasman, Anders Gorm Pedersen, Frank Møller Aarestrup, Ole Lund

**Affiliations:** 1 Center for Biological Sequence Analysis, DTU Bioinformatics, Technical University of Denmark, Kongens Lyngby, Denmark; 2 Department of Microbiology and Infection Control, Statens Serum Institute, Copenhagen, Denmark; 3 Research Group for Genomic Epidemiology, DTU FOOD, Technical University of Denmark, Kongens Lyngby, Denmark

**Keywords:** Phylogeny, Evolution, Benchmark, WGS

## Abstract

**Background:**

Whole genome sequencing (WGS) is increasingly used in diagnostics and surveillance of infectious diseases. A major application for WGS is to use the data for identifying outbreak clusters, and there is therefore a need for methods that can accurately and efficiently infer phylogenies from sequencing reads. In the present study we describe a new dataset that we have created for the purpose of benchmarking such WGS-based methods for epidemiological data, and also present an analysis where we use the data to compare the performance of some current methods.

**Results:**

Our aim was to create a benchmark data set that mimics sequencing data of the sort that might be collected during an outbreak of an infectious disease. This was achieved by letting an *E. coli* hypermutator strain grow in the lab for 8 consecutive days, each day splitting the culture in two while also collecting samples for sequencing. The result is a data set consisting of 101 whole genome sequences with known phylogenetic relationship. Among the sequenced samples 51 correspond to internal nodes in the phylogeny because they are ancestral, while the remaining 50 correspond to leaves.

We also used the newly created data set to compare three different online available methods that infer phylogenies from whole-genome sequencing reads: NDtree, CSI Phylogeny and REALPHY. One complication when comparing the output of these methods with the known phylogeny is that phylogenetic methods typically build trees where all observed sequences are placed as leafs, even though some of them are in fact ancestral. We therefore devised a method for post processing the inferred trees by collapsing short branches (thus relocating some leafs to internal nodes), and also present two new measures of tree similarity that takes into account the identity of both internal and leaf nodes.

**Conclusions:**

Based on this analysis we find that, among the investigated methods, CSI Phylogeny had the best performance, correctly identifying 73% of all branches in the tree and 71% of all clades.

We have made all data from this experiment (raw sequencing reads, consensus whole-genome sequences, as well as descriptions of the known phylogeny in a variety of formats) publicly available, with the hope that other groups may find this data useful for benchmarking and exploring the performance of epidemiological methods. All data is freely available at: https://cge.cbs.dtu.dk/services/evolution_data.php.

**Electronic supplementary material:**

The online version of this article (doi:10.1186/s12864-016-3407-6) contains supplementary material, which is available to authorized users.

## Background

The ability to detect and track outbreaks of infectious diseases is of vital importance to maintain public health. The advances of Next Generation Sequencing (NGS) technology has led to decreasing cost and growing speed of Whole Genome Sequencing (WGS) [1]. Due to this, the technology has gained increasing importance in routine clinical microbiology and for studying and detecting outbreaks and epidemics [2–4]. Various studies have shown that inference of the phylogenetic relationship between WGS isolates is helpful for determining epidemiological relationships [5, 6], and a number of methods for inferring phylogenies directly from NGS data have been created. Methods available online which accept raw reads data include snpTree [7], NDtree [8, 9] and CSI Phylogeny [10] available from Center for Genomic Epidemiology. Furthermore REALPHY from the Swiss Institute of Bioinformatics is also online available and can be downloaded for local installation [11]. In addition to this many groups are building in-house pipelines for outbreak detection.

There were two main goals of the present study: (1) to create a data set that could be used to benchmark NGS-based methods for epidemiological data, and (2) to use this for comparing the performance of some current methods. We wanted the benchmark data set to mimic NGS data of the sort that might be collected during an outbreak of an infectious disease. This was achieved by letting an *E. coli* hypermutator strain grow in the lab for 8 consecutive days. Each day all growing cultures were divided in two, and samples were taken for sequencing. The result was a total of 255 samples corresponding to both internal (ancestral) and external (leaf) nodes on a bifurcating phylogenetic tree.

To the best of our knowledge there is currently no other large scale in vitro WGS data sets with known phylogeny for evaluation of WGS phylogeny methods, and it is our hope that this data will prove useful for benchmarking and optimization of future methods. The group of Richard Lenski at Michigan State University has performed a long-term experimental evolution project, that has now been running since 1988 [12, 13], and which might also be useful for this purpose, although only a limited number of full genome sequences are so far available.

## Results

### *Escherichia coli* hypermutator strain

To ensure a measureable difference between each sequenced sample in the data set, the experiment was set up to give a high probability of observing at least one mutation between each sequenced culture sample. Wild type *E. coli* has a mutation rate around 10^−3^ mutations per genome per generation [14] corresponding to about 0.05 mutations per genome per day at a generation time of 30 min [15]. At this rate each sample would have to grow for 20 days to undergo an average of one mutation per genome. The *E. coli* hypermutator strain CSH114, on the other hand, has been reported to have a mutation rate that is about 100–1000 fold higher due to a mutation in the *mutT* gene which makes it prone to AT → GC mutations [14, 16]. Using an assay based on the frequency of spontaneous development of Rifampicin resistance (see Methods), we estimated the mutation rate of the hypermutator strain to be about 160 times higher than a wild type *E. coli*. At the reported generation time of 44 min for CSH114, this corresponds to an average of about 5 mutations per day, which is in a suitable range for our purposes, and we therefore proceeded to use this strain for our in vitro evolution experiment.

### In vitro evolution experiment

**Fig. 1:**
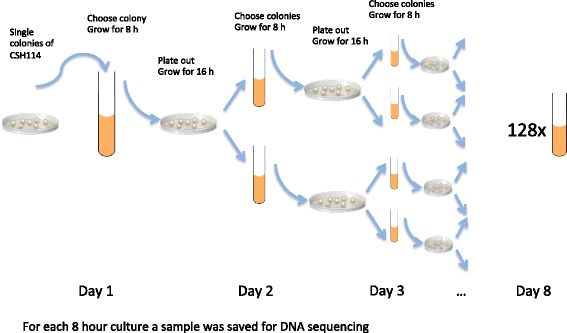
Setup for the in vitro evolution experiment*.* Each day two single colonies were transferred to 20 mL LB broth, to grow for 8 h. 1 μL of culture was plated on LB plate for overnight growth. This continued for 8 days until 128 tubes of culture was obtained, and 255 samples had been saved for DNA sequencing

**Fig. 2:**
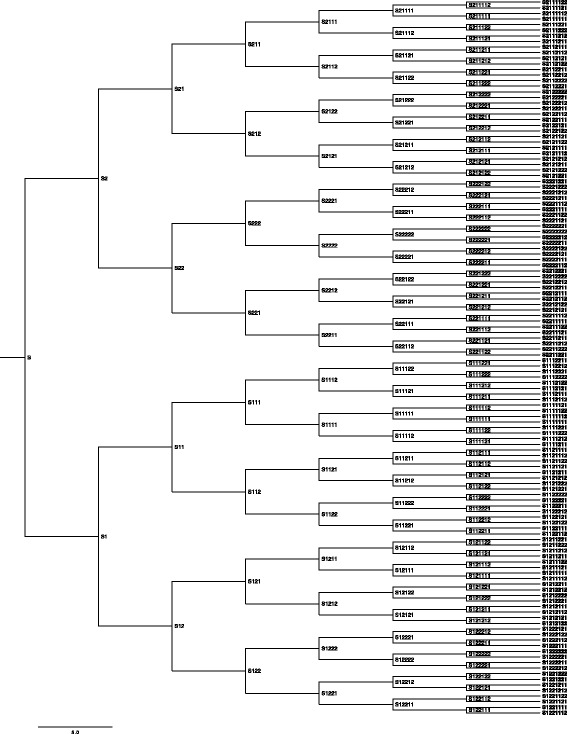
Ideal tree. This tree shows the expected phylogeny of the in vitro evolution experiment, with all 255 strains indicated as either tips or ancestral nodes

The main idea of the in vitro evolution experiment was to start with a single colony of *E. coli* CSH114 *mutT*, which after 8 days of growth and daily division of cultures would give rise to 128 related, but diverged, populations. Specifically, each 24-hour cycle in our experiment consisted of the following steps (Fig. 1): (1) Streaking to single colonies, followed by 16 h of growth on plate. (2) Inoculation of a single colony from the plate followed by 8 h of growth in liquid culture. (3) Isolation of a sample for sequencing. (4) Repeating the procedure from step 1. Starting from the second of these 24-hour cycles two colonies were picked from each plate, resulting in a splitting of the original population, and a daily doubling of the number of growing cultures. On consecutive days we therefore collected 1, 2, 4, 8, 16, 32, 64, and 128 culture samples for sequencing respectively, resulting in a total of 255 samples. From these 255 samples, we obtained whole genome sequences from 101 (see Methods). The 101 sequenced samples came from all 8 levels in the tree, and corresponded to both external (leaf) and internal (ancestral) nodes. The tree showing the real, known relationship between the samples is shown in Fig. 2. Note that we employed a naming convention where the original single colony sequence was named S; its two descendants were named S1, and S2; each of their two descendants were named S11, S12, and S21, S22, respectively, *etc*., *etc*.

**Fig. 3:**
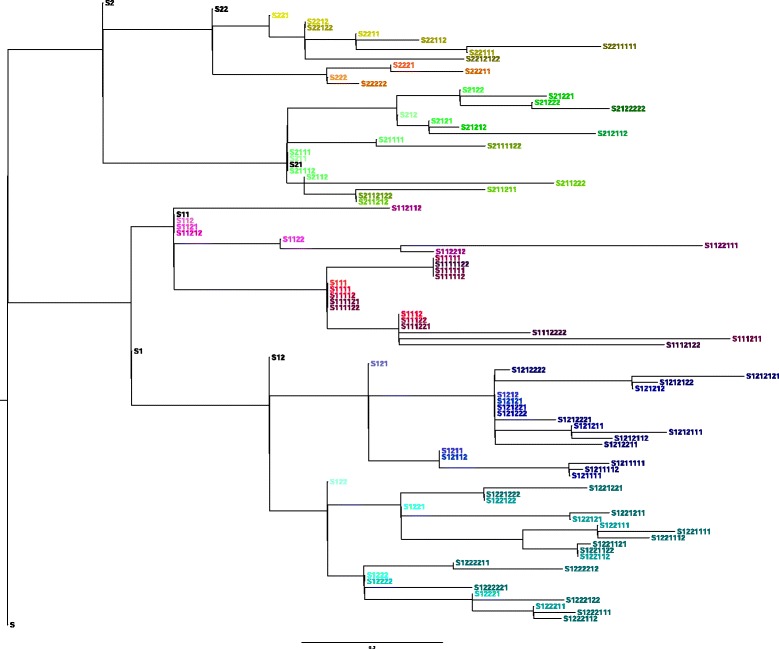
Phylogenetic tree inferred by CSI phylogeny. This is the un collapsed version of the best scoring tree according to the new method for tree comparison. The phylogeny is inferred by CSI Phylogeny on all 101 sequenced strains, using the assembled contigs from the root strain as reference genome, SNP pruning disabled

All data from this experiment (raw sequencing reads, consensus sequences obtained by mapping to the reference genome NC_000913, as well as descriptions of the known phylogeny in a variety of formats) has been made publicly available at the following website: https://cge.cbs.dtu.dk/services/evolution_data.php. It is our hope that other groups may find this data useful for benchmarking and exploring the performance of epidemiological methods.

Note that individual bacteria in the growing colonies and liquid cultures are accumulating mutations constantly through the daily cycle, and the sample taken for sequencing each day therefore consists of a diverse population. Specifically, each genome in this population will have gained its own set of (on average) about 5 mutations compared to the founding single cell from the original streaking. However, when we derive a single whole genome consensus sequence based on the reads obtained from such a sample, we expect to retrieve the sequence of the original single cell’s genome. This is because only a very low fraction of bacteria will have experienced a change at any specific nucleotide position, and the vast majority of reads mapped at that location will therefore have the original nucleotide. (Specifically, a rate of 5 substitutions/genome/day corresponds to a rate of about 10^−6^ substitutions/site/day, and hence only 1 read in a million is expected to have a mutation at any specific site). Populations with new consensus sequences, an average of 5 mutations separated from their ancestor, are created by the “founder effect” that occurs when we streak to single colonies anew.

### Benchmarking of phylogenetic methods for whole-genome, epidemiologic NGS data

In addition to creating a benchmark data set as described above, we were also interested in assessing the performance of some current epidemiological phylogenetic methods that infer phylogenies from NGS data. Specifically, we used the following three methods to analyze our newly created dataset: CSI Phylogeny [10], NDtree [8, 9] and REALPHY [11].

We used each of the three methods to infer phylogenies from all 101 sets of whole genome sequencing reads (resulting in trees with 101 leaves). For each method we furthermore explored a number of settings (Table 1): First, we explored the impact of using different reference genomes for mapping reads. The investigated references genomes had differing degrees of similarity to the mapped reads. In order of increasing distance the investigated reference genomes were: (1) de novo assembled contigs from the root strain S (very close); (2) *E. coli* K-12 MG1655 (NC_000913; close); (3) *E. coli* K-12 BW2952 (NC_012759; close); and (4) *E. coli* UMNK88 (NC_017641; distant).

**Table 1:** Methods, thresholds and reference genomes for inference of phylogeny for all 101 sequenced strains

Method	Tree name	Reference genome	Threshold 1	Threshold 2	Tree method
CSI Phylogeny	CSI_all_1	Assembled contigs from root sample	Z-score 1.96	Prune disabled	FastTree
CSI Phylogeny	CSI_all_2	Assembled contigs from root sample	Z-score 1.96	Prune set to 10	FastTree
CSI Phylogeny	CSI_all_3	*E. coli* NC_000913	Z-score 1.96	Prune disabled	FastTree
NDtree	ND_all_1	Assembled contigs from root sample	Z-score 1.96	X 10 x < Y	Neighbor Joining
NDtree	ND_all_2	Assembled contigs from root sample	Z-score 1.96	X 10 x < Y	UPGMA
NDtree	ND_all_3	Assembled contigs from root sample	Pairwise comparison	X 10 x < Y	Neighbor Joining
NDtree	ND_all_4	Assembled contigs from root sample	Z-score 1.64	X 10 x < Y	Neighbor Joining
NDtree	ND_all_5	*E. coli* NC000913	Z-score 1.96	X 10 x < Y	Neighbor Joining
NDtree	ND_all_6	*E. coli* NC012759	Z-score 1.96	X 10 x < Y	Neighbor Joining
NDtree	ND_all_7	*E. coli* NC017641	Z-score 1.96	X 10 x < Y	Neighbor Joining
Realphy	RP_all_1	*E. coli* NC012759 and *E. coli* NC000913	Weight ≥ 10	≥95% supports the same nucleotide	RAxML
Realphy	RP_all_2	*E. coli* NC012759 and *E. coli* NC000913	Weight ≥ 10	≥ 95% supports the same nucleotide	phyML
Realphy	RP_all_3	*E. coli* NC012759, *E. coli* NC000913, *E. coli* NC017641	Weight ≥ 10	≥ 95% supports the same nucleotide	phyML

For the CSI Phylogeny method we furthermore explored the effect of cutoffs for filtering data. This method maps reads to the given reference genomes and filters SNPs based on their quality, using a Z-score cutoff, which is used to determine if X is significantly larger than Y (here a cutoff of 1.96 was used). The CSI Phylogeny method can also filter SNPs from the analysis by a process called pruning. The default setting is to remove SNPs such that no SNPs are within 10 base pairs of each other. In the present analysis we explored the impact of disabling pruning, thus including all SNPs in the analysis. CSI Phylogeny uses the FastTree method to build the trees. FastTree is a method that infers approximate maximum likelihood trees, and which can handle very large alignments.

The NDtree method for inferring phylogeny splits the raw reads to k-mers and maps them to the reference genome. Based on this an ungapped consensus sequence with the same length as the reference genome is created; the differences between the consensus sequences are counted and used as the phylogenetic distance. The Z-score is used to evaluate the base calling, the higher, the stricter. The “pairwise comparison” threshold is where all positions that are called in both sequences of a pair to be compared are used, instead of only using positions that were significant in all sequences. This has the advantage that more positions can on the average be used to compare sequences, but the disadvantage that different sets of positions are used for comparing different pairs of sequences. NDtree uses either UPGMA or neighbor joining to build trees from the estimated distance matrix. UPGMA assumes that all leaves in a tree have the same distance from the root (i.e, that the substitution rate on all branches is identical). This assumption can be problematic if some observed sequences in fact correspond to internal nodes. Neighbor joining on the other hand does allow for different rates on different branches.

The REALPHY method has two standard thresholds for trusting the base call. The first is that the weight of the mapping has to be higher than 10, the second is that 95% of the mappings has to support the same nucleotide. REALPHY uses either phyML or RAxML, both of which are fast maximum likelihood methods.

The method which comes closest to infering the known phylogeny is CSI Phylogeny with SNP pruning disabled and the assembled contigs from the root sample as reference genome (Fig. 3). The other infered trees can be found in Additonal files 1, 2, 3, 4, 5, 6, 7, 8, 9 and 10. Trees with bootstrap values can be found in Additonal files 11, 12 and 13. Additional files 14, 15, 16, 17, 18, 19 and 20 contain SNP alignments and positions for the inferred phylogenies.

**Fig. 4:**
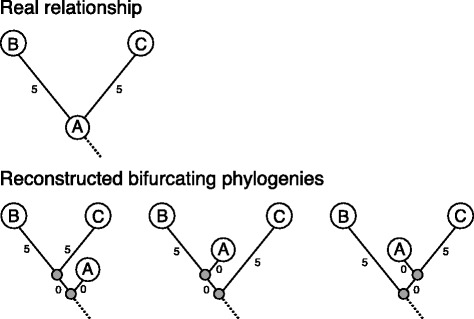
Tree building artifacts, when constructing bifurcating trees with ancestral nodes. This figure shows the three possible resolutions of an ancestral node in a bifurcating tree, which only allows leaves in the tree

**Fig. 5:**
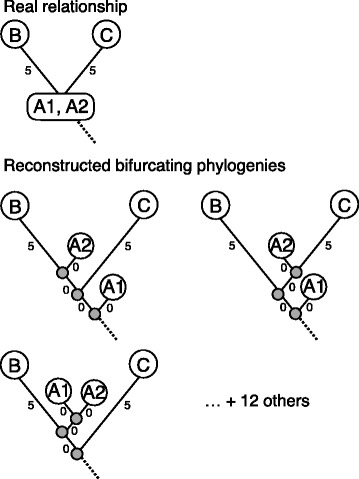
Tree building artifacts, when constructing bifurcating trees on identical sequences. This figure shows 3 of the 15 possible resolutions for building a bifurcating tree with two identical sequences as possible ancestral nodes

An important point is that the benchmark data set analyzed here includes several sequences that are (directly or indirectly) ancestral to other sequences in the data set. The real relationship between the observed sequences (shown in Additional files 21 and 22) is therefore one where some sequences correspond to internal nodes in the tree, while others correspond to leaves. However, the methods we investigate here (like most other phylogenetic methods) do not explicitly take this into account, and they therefore instead produce trees where all observed sequences are placed as leaves. This causes problems when one wants to compare the reconstructed phylogenies to the known, real phylogeny or to each other. Specifically, what typically happens, when standard phylogenetic methods are used on epidemiological data, is that ancestral sequences, which ought to be located at internal nodes in the tree, will instead be attached as leaves on very short (maybe even zero-length) branches close to the internal nodes where they belong. (In fact, a tree where an ancestral sequence is placed as a leaf will require *two* branches extra compared to a tree where the ancestral sequence is instead placed at an internal node). As it turns out, on a rooted, bifurcating tree there are three different ways an ancestral sequence can be placed as a leaf next to the internal node where it rightfully belongs (Fig. 4). Judged on the criteria used for both likelihood- and distance-based phylogenetic methods respectively, these three alternative ways of placing the ancestral sequence will all be equally good, and will furthermore be (nearly) as good as the real tree, at least if the two extra branches have (nearly) zero length. In the case of distance-based methods such as neighbor joining, this is because, for all three trees, the pairwise distances between taxa measured along the branches of the tree (the patristic distance) will match the pairwise distances between sequences (the distance matrix) equally well, since the two additional, short branches have very little impact on these. Likelihoods will also be identical or almost identical for the three possible alternative trees (and the real tree), since there is probability near 1 of having the same nucleotide at either end of a very short branch, and multiplying by this will not change the overall likelihood much. Consequently, different phylogenetic methods may choose either of three ways of placing an ancestral sequence as a leaf depending on arbitrary and possibly random criteria. Since the placement of an ancestral sequence as a leaf near any given internal node is independent of how ancestral sequences are placed near other internal nodes, the total number of possible, equally good resolutions is 3 raised to the power of the number of internal nodes. (For instance, in a tree with 127 internal nodes - such as the real relationship between our 255 sequences - there are 3^127^ = 3.9*10^60^ possible, equally good bifurcating resolutions of the real, ancestral tree). It is therefore not meaningful to assess the reconstructed phylogenies by directly using measures of tree-distance that rely on branching order in the trees (such as the frequently used Robinson-Foulds’ distance [17]): there are so many possible ways of placing ancestral sequences as leaves that even two resolved trees, that in principle agree completely on the underlying ancestral tree, might have almost zero similarity. Indeed, preliminary attempts to use the Robinson-Foulds’ measure to assess the correctness of the trees by comparing to a randomly resolved version of the real tree, showed very large distances (data not shown). The problem described above is exacerbated if an observed sequence is found to be exactly identical to another observed sequence (as might happen in our case if zero mutations have accumulated after a day’s growth): in this case, the real relationship would be one where an internal node in the tree corresponded to *two* observed sequences, and here there would be 15 different, possible bifurcating resolutions where the internal nodes were placed as leaves by adding short, or zero length branches (Fig. 5; 15 is the number of possible rooted, bifurcating trees with 4 leaves).

**Fig. 6:**
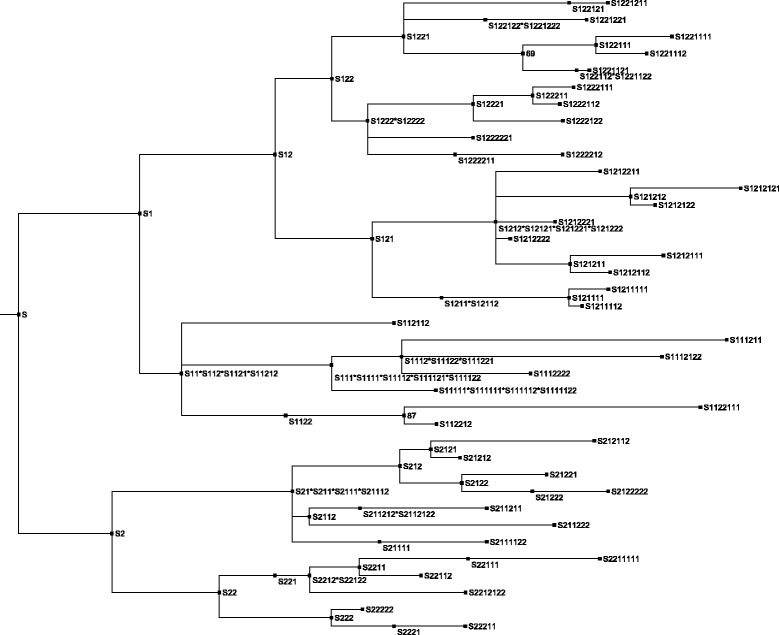
Collapsed bifurcating tree from CSI phylogeny. This is the collapsed version of the best scoring tree according to the new method for tree comparison. The tree was collapsed to have 41 remaining leaves. The phylogeny is inferred by CSI Phylogeny on all 101 sequenced strains, using the assembled contigs from the root strain as reference genome, SNP pruning disabled

At the same time, manual inspection of the reconstructed phylogenies clearly indicated that the trees captured many aspects of the real relationship: typically, sequences with the same name prefix (e.g., S11, S111, S112, S1111, S1112, S1121, etc.) were found to be in the same sub-tree as expected (since longer names with the same prefix are descendants of the sequence with the shortest name). We therefore developed what we deem to be a more meaningful way of measuring the correctness of these trees. Our solution has two parts: (1) We constructed an algorithm for collapsing short branches on trees, such that a sequence located at the end of a collapsed branch (e.g., as a leaf) is instead placed together with its own ancestral node. In this way we can interpret the reconstructed phylogenies as if some of the observed sequences were in fact ancestral. (2) We devised two new measures of tree similarity that specifically take into account the identity of both the parent and the child node on a branch (unlike measures such as Robinson-Foulds’ which only takes into account leaf sets, in the form of tree bipartitions, and do not directly take internal node identities into account). We then used these measures to compare the collapsed versions of the trees with the known, real phylogeny.

With regard to the algorithm for collapsing branches, we used two different approaches: in one approach, we used a predetermined branch-length cutoff to decide whether or not a branch should be collapsed. In the present case we collapsed branches with a length that was less than or equal to 0.0 (in distance-based trees, negative branch-lengths occasionally occur). In the second approach we instead sorted all branch lengths in the tree, and then tried using increasingly larger values from this list as cutoffs until a desired number of leaves (or less) was left in the collapsed tree. In the present case we used 50 as the target value, since that was the known number of leaves in our benchmark data (which had a total of 101 sequences); the cutoffs used for optimization and the remaining number of tips can be seen in the Additional files 23, 24 and 25. If several consecutive branches were collapsed, then this resulted in the creation of internal nodes with > = 3 names.

The two tree-similarity measures we suggest are the following: (1) The percentage of correct parent-child relationships. The main idea in this measure is to describe a rooted tree as a list of parent-child relationships, where parent and child means the names of the sequences at the two ends of a branch (and the parent is the node closest to the root). A collapsed tree can then be compared to a benchmark (or to another collapsed tree) by computing the fraction of parent-child relationships that are identical in the two trees. (2) The percentage of correct clades. In this measure, we, for each internal node in a tree write a list of all its descendants (the clade rooted at that internal node, where we in this case also include internal nodes among the descendants). This measure is related to the parent-child relationship measure but is not necessarily identical (it is possible to have a perfectly matching parent-child relationship for a given internal branch, but not having all the same descendants further downstream). Again, we use this measure to compare a collapsed tree to the benchmark, by computing the fraction of clades in the benchmark that are also present in the investigated tree. An advantage of the suggested measures compared to Robinson-Foulds’ distance is that they are on a more naturally interpretable scale (0–100% identity). Our clade-based measure is actually identical to the distance measure originally proposed by Robinson and Foulds, where internal nodes could also be labeled, but is different from the implementations typically found which only rely on sets of leaf names.

The results of these comparisons can be seen in Table 2 (also see Additional file 23). The main observations are as follows: CSI phylogeny (Fig. 6) with the disabled SNP pruning was able to infer 73% of the parent child relations and 71% of the clade structure. The NDtree method was able to infer 65% parent child relations and 63% of the clades structure, with the default settings, the Neighbor Joining tree algorithm and the reference genome was not important. REALPHY using phyML, was able to infer 55% of the parent child relations and 51% of the clade structure.

**Table 2:** Comparisons of reconstructed phylogenies to the known topology of the dataset

Tree method	Tree name	Fraction of correct parent/child relations	Fraction of correct clades in tree structure
CSI phylogeny, pruning disabled	CSI_all_1	0.73	0.71
CSI phylogeny, pruning set to 10 bp	CSI_all_2	0.52	0.61
CSI phylogeny, pruning disabled NC_000913 as ref	CSI_all_3	0.52	0.61
NDtree, z-score 1.96	ND_all_1	0.65	0.63
NDtree, z-score 1.96, UPGMA tree method	ND_all_2	0.20	0.08
NDtree pairwise comparison, z-score 1.96	ND_all_3	0.26	0.29
NDtree, z-score 1.64	ND_all_4	0.65	0.63
NDtree, NC_000913 as ref, z-score 1.96	ND_all_5	0.65	0.63
NDtree, NC_012759 as ref, z-score 1.96	ND_all_6	0.65	0.63
NDtree, NC_017641 as ref, z-score 1.96	ND_all_7	0.65	0.63
REALPHY, ref NC_012759 and NC_000913, PhyML	RP_all_1	0.55	0.51
REALPHY, ref NC_012759 and NC_000913, RAxML	RP_all_2	0.33	0.24

### Analysis of mutation rates

**Fig. 7:**
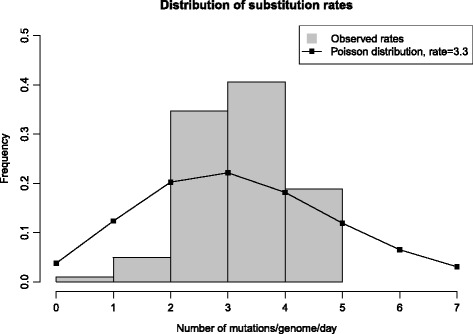
Histogram. Number of mutations per day as estimated by directly comparing each genome sequence to the sequence of the day 1 isolate, S, and dividing by the age difference

The full genome sequences of all 101 strains were used to estimate the average substitution rate. For each isolate, we counted the total number of nucleotide positions having a different nucleotide than isolate S (which is the isolate closest to the root), and then divided these numbers by the isolate’s age in days to give the observed number of mutations per genome per day. The value estimated in this manner was 3.3 mutations per genome per day, i.e. slightly less than the expected value of about 5 estimated from the Rifampicin assay (but well within the uncertainty of that analysis). Figure 7 shows a comparison between the distribution of observed rates and a Poisson distribution with rate 3.3. It can be seen that there is somewhat less variation in the observed distribution compared to a Poisson distribution (the data is “underdispersed”).

We also estimated the substitution rate using the software BEAST (Bayesian Evolutionary Analysis Sampling Trees), using the known sampling days (“dated tips”) for calibrating the rate estimation [18]. The analysis was performed on an alignment of the 392 variable sites identified based on the pairwise NDtree analysis. Based on this analysis the mean rate was estimated at 2.8 substitutions per genome per day (posterior mean), with a 95% credible interval of 2.5 to 3.2 substitutions per genome per day. This corresponds nicely to the values reported above.

### Branch support for reconstructed tree topologies

To investigate the confidence of the tree topology, bootstrap values were analyzed. Trees produced by the neighbor package have not been bootstrapped; therefore FastTree was used to infer a tree with bootstrap values on the SNP alignment from NDtree using the de novo assembled contigs from day 1 as reference genome. PhyML produced a tree with bootstrap values for REALPHY. FastTree produced a tree with bootstrap values for CSI Phylogeny. In all three trees approximately 60% of the internal nodes had a bootstrap confidence interval above 90%. The bootstrapped trees are found in Additional files 11, 12 and 13.

## Discussion

Rapid and reliable identification of infectious disease clusters is essential to guide outbreak response and control measures. Next-generation sequencing shows great promise to improve the routine characterization of infectious disease agents in microbial laboratories and sequencing data are attractive because they both provide high resolution as well as a standardized data format (the DNA sequence) that may be exchanged and compared between laboratories and over time. However, if different laboratories use different methods for building phylogenies and thus, identify outbreak clusters this may create unnecessary discussions and delays.

To our knowledge we are the first to create a WGS dataset with known phylogeny that can be used to benchmark whole genome phylogenetic and epidemiological methods. We have made all of our data available online, with the hope that other researchers can used them for investigating and improving the performance of existing methods. A summation of the known relationship is found in Additional file 26.

In our findings we see similarities to the results from Hillis et al. [19], such as the fact that the UPMGA method is not able to correctly infer phylogeny of samples that have unequal evolution rates, or have been sampled at different times. There are also many differences between Hillis et al. [19], and this study. First of all, this study uses WGS data and not restriction site maps, this means that there is a lot of emphasis on finding the correct SNPs, as well as inferring the phylogeny from these. Second of all, in Hillis et al. [19] they know the full knowledge of all the mutations in the restrictions sites, as well as the known topology, as they could measure the responses to all restriction enzymes. In this study, the full truth of all mutations is not known, only the structure of the experiment is known and therefore the topology is known.

## Conclusion

In this study we have succeeded in making a data set with known phylogeny and made it publicly available. We used this as a benchmark data set to assess the performance of a number of freely available phylogenetic analysis pipelines. The main conclusion is that it was possible to obtain up to 73% of the known phylogeny, by using CSI Phylogeny with a closely related reference genome and no SNP pruning. Furthermore the other methods were able to reconstruct more than 50% of the phylogeny given the right settings.

## Methods

### Rifampicin plate-assay

In order to estimate the mutation rate of the CSH114 strain compared to a similar non-hypermutator *E. coli* strain (*Escherichia coli* CGSC3004 [16]), the strains were tested for the frequency with which Rifampicin resistance developed after 8 h of growth in LB broth (salt concentration 5 g/L) at 37 °C with 80 rpm shaking. Subsequently, 800 μL CGSC3004 culture and 100 μL CSH114 culture was plated on brain heart infusion (BHI) agar plates with 25 μg/mL rifampicin, 4 plates for each strain, 8 plates in total. After O/N growth at 37 °C, the number of single colonies was counted. As a control it was verified that strains grew normally on BHI agar without rifampicin. Table 3 shows the raw counts of the single colonies. As 8 times more culture was used for the non-hypermutator strain, the results indicate a substitution rate for the *mutT* strain CSH114 that on average is increased 164-fold compared to the non-mutator strain. Based on the previously reported substitution rate of about 10^−3^ mutations per genome per generation for the non-mutator *E. coli* [14], and a generation time of 44 min for the *E. coli* CSH114 mutator strain [20], we can estimate that CSH114 will have a substitution rate of about 5.4 mutations per genome per day.

**Table 3:** The number of single colonies on the plates from the Rifampicin plate-assay

	CGSG3004	CSH114
	9	170
	6	240
	15	180
	5	148
Average	8.8	185

### In vitro evolution

At the start of the experiment (day 0), CSH114 was streaked on LB plates and grown for 16 h at 37 °C. On day 1, a single colony was inoculated in 20 mL LB broth and incubated at 37 °C with 80 rpm shaking. After 8 h 1 mL of culture was saved for sequencing. Hereafter, a 1 μL loop was used to streak the culture onto an LB plate, which was incubated at 37 °C for 16 h. From this point on, the following 24-hour cycle was repeated until 8 days: (1) streaking to single colonies, (2) 16 h growth on LB plate, (3) inoculation of two single colonies in liquid LB broth, (4) 8 h growth in LB broth, (5) sequencing sample, repeat.

### Whole genome sequencing

One milliliter of culture, from every 8-hour culture, was spun down and the pellet was diluted in 200 μL PBS buffer (Invitrogen, Carlsbad, CA). The buffer and pellet was frozen and later used for DNA sequencing. The genomic DNA was isolated using the Easy-DNA isolation kit (Invitrogen, Carlsbad, CA). DNA concentration was measured by Qubit dsDNA (double-stranded DNA) BR and HS assay kits (Invitrogen). 101 of the 255 samples had a sufficiently high DNA concentration for whole genome sequencing. DNA libraries were built using Nextera XT (Illumina), and sequenced by Illumina MiSeq (Illumina) to a minimal coverage of 30×.

### Phylogenetic methods

**CSI phylogeny** 1.2 is available online at https://cge.cbs.dtu.dk/services/CSIPhylogeny-1.2/ [10]. CSI Phylogeny 1.2, uses BWA version 0.7.12 [21], SAMtools version 0.1.18 [22], BEDtools version 2.16.2 [23], MUMmer version 3.23 [24] and FastTree version 2.1.7 [25].

**NDtree** 1.2 is available online at https://cge.cbs.dtu.dk//services/NDtree/ and for local installation from https://bitbucket.org/genomicepidemiology/assimpler [8, 10]. Besides from using in-house scripts found in the bitbucket folder, NDtree uses the Neighbor program from the Phylip package version 3.695 [26].

**REALPHY** version 1.12 is available for download and local installation from http://realphy.unibas.ch/fcgi/realphy [11]. The local installation used Bowtie2 version 2.2.4 [27], phyML version 3.1 [28], RAxML version 8.2.4 [29].

### Tree comparison

The author’s own scripts and libraries were used for collapsing short branches, as well as for computing percentage correct parent-child relationships, and percentage of correct descendant clades.

#### BEAST

We used BEAUti and BEAST version 2.4.3 to estimate substitution rates from an alignment of variable sites (SNPs identified by the NDtree method). Settings were as follows: Sequences were annotated with their known sampling day (“dated tips”). Substitution model: TN93 + gamma. Prior on clock rate: both lognormal and gamma priors with different widths and centers were explored (and different settings were found not to have much impact on the results). Priors on kappa parameters: wide lognormal distributions. Popsize prior: 1/X. Tree prior: both coalescent exponential population and coalescent constant population were explored and found not to have a major impact on estimated rates (estimated tree heights did differ slightly between the two: 8.6 days for constant, and 7.7 days for exponential; both of these estimates still correspond nicely to the experimental setup running over 8 days). MCMC was run for 10,000,000 iterations. Convergence was checked by inspecting effective sample sizes (ESS) and parameter value traces in the software Tracer (version 1.6.0), and by ensuring that similar posterior distributions were obtained in several independent runs. Clock rate estimates from BEAST were in substitutions per site per day, and were multiplied by alignment length (392 sites) to get the rate per genome per day.

## Additional files

Additional file 1:
**a** Tree inferred by CSI Phylogeny pruning set to 10, contigs from day 1 used as reference. PDF. **b** Tree inferred by CSI Phylogeny pruning set to 10, contigs from day 1 used as reference. Newick file. (ZIP 6 kb)
Additional file 2:
**a** Tree inferred by CSI Phylogeny pruning disabled, *E. coli* MG1655 (NC_000913) used as reference. PDF. **b** Tree inferred by CSI Phylogeny pruning disabled, *E. coli* MG1655 (NC_000913) used as reference. Newick file. (ZIP 6 kb)
Additional file 3:
**a** Tree inferred by NDtree, Z-score 1.96, Neighbor Joining tree, contigs from day 1 used as reference. PDF. **b** Tree inferred by NDtree, Z-score 1.96, Neighbor Joining tree, contigs from day 1 used as reference. Newick file. (ZIP 5 kb)
Additional file 4:
**a** Tree inferred by NDtree, Z-score 1.64, Neighbor Joining tree, contigs from day 1 used as reference. PDF. **b** Tree inferred by NDtree, Z-score 1.64, Neighbor Joining tree, contigs from day 1 used as reference. Newick file. (ZIP 5 kb)
Additional file 5:
**a** Tree inferred by NDtree, Z-score 1.96, Neighbor Joining tree, *E. coli* MG1655 (NC_000913) used as reference. PDF. **b** Tree inferred by NDtree, Z-score 1.96, Neighbor Joining tree, *E. coli* MG1655 (NC_000913) used as reference. Newick file. (ZIP 5 kb)
Additional file 6:
**a** Tree inferred by NDtree, Z-score 1.96, Neighbor Joining tree, *E. coli* NC_012759 used as reference. PDF. **b** Tree inferred by NDtree, Z-score 1.96, Neighbor Joining tree, *E. coli* NC_012759 used as reference. Newick file. (ZIP 5 kb)
Additional file 7:
**a** Tree inferred by NDtree, Z-score 1.96, Neighbor Joining tree, *E. coli* NC_017641 used as reference. PDF. **b** Tree inferred by NDtree, Z-score 1.96, Neighbor Joining tree, *E. coli* NC_017641 used as reference. Newick file. (ZIP 5 kb)
Additional file 8:
**a** Tree inferred by REALPHY, PhyML, NC_000913, NC_012759 and NC_017641 used as reference. PDF. **b** Tree inferred by REALPHY, PhyML, NC_000913, NC_012759 and NC_017641 used as reference. Newick file. (ZIP 6 kb)
Additional file 9:
**a** Tree inferred by REALPHY, PhyML, NC_000913 and NC_012759 used as reference. PDF. **b** Tree inferred by REALPHY, PhyML, NC_000913 and NC_012759 used as reference. Newick file. (ZIP 5 kb)
Additional file 10:
**a** Tree inferred by REALPHY, RAxML, NC_000913 and NC_012759 used as reference. PDF. **b** Tree inferred by REALPHY, RAxML, NC_000913 and NC_012759 used as reference. Newick file. (ZIP 6 kb)
Additional file 11: Tree inferred by FastTree on the alignment from the pairwise NDtree analysis. Bootstrap values shown. No isolate names. (PDF 4 kb)
Additional file 12: Tree inferred by CSI Phylogeny, same tree as Fig. 3. Bootstrap values shown. No isolate names. (PDF 20 kb)
Additional file 13: Tree inferred by FastTree on the alignment from the REALPHY phyML analysis with 2 reference genomes. Bootstrap values shown. No strain names. (PDF 4 kb)
Additional file 14: SNP alignment from the NDtree analysis with contigs from day 1 as reference genome. (FASTA 40 kb)
Additional file 15: Position for mutations from S14 SNP alignment. (TXT 2 kb)
Additional file 16: SNP alignment from the NDtree analysis with NC_000913 as reference genome. (FASTA 40 kb)
Additional file 17: Position for mutations from S16 SNP alignment. (TXT 2 kb)
Additional file 18: SNP alignment from the CSI Phylogeny analysis with NC000913 as reference genome. (FASTA 44 kb)
Additional file 19: SNP alignment from the REALPHY phyML analysis with NC000913 and NC012759 as reference genomes. (FASTA 85 kb)
Additional file 20: Position for mutations from S19 SNP alignment. Positions related to NC000913. (TXT 5 kb)
Additional file 21: Spreadsheet with an overview of all 255 strains from the in vitro evolution experiment. The sequenced strains are marked in bold. (XLSX 29 kb)
Additional file 22: The benchmark child list with the known tree structure for the experiment, which all of the trees inferred on all the 101 strains was compared to. Tab separated. (TXT 1 kb)
Additional file 23: Tab separated table showing all results from the new tree comparison method, where matching parent/child relations and matching clades to the benchmark child list are counted. The cutoff branch length is optimized. (TXT 6 kb)
Additional file 24: The full results from the cutoff optimization. (TXT 6 kb)
Additional file 25: Tab separated table, showing results from the new tree comparison method when the cutoff was set to 0. (TXT 2 kb)
Additional file 26: Tab separated table with systematic names, fastq filenames, age in the experiment and expected mutations to the strain from day 1 of the experiment, and indication of whether the node is a tip or internal node in the topology. (TXT 3 kb)

## Abbreviations

BEAST: Bayesian Evolutionary Analysis Sampling Trees
BHI: Brain-heart infusion
bp: Base pairs
NDtree: Nucleotide Difference tree
NGS: Next Generation Sequencing
SNP: Single Nucleotide Polymorphism
UPGMA: Unweighted Pair Group Method with Arithmetic Mean
WGS: Whole Genome Sequencing
