# Supplementary figures and images for: Bacterial whole genome-based phylogeny: construction of a new benchmarking dataset and assessment of some existing methods
Source: BMC Genomics. 2017 Jan 5;18:19. doi: 10.1186/s12864-016-3407-6 (PMC5217230; doi:10.1186/s12864-016-3407-6)

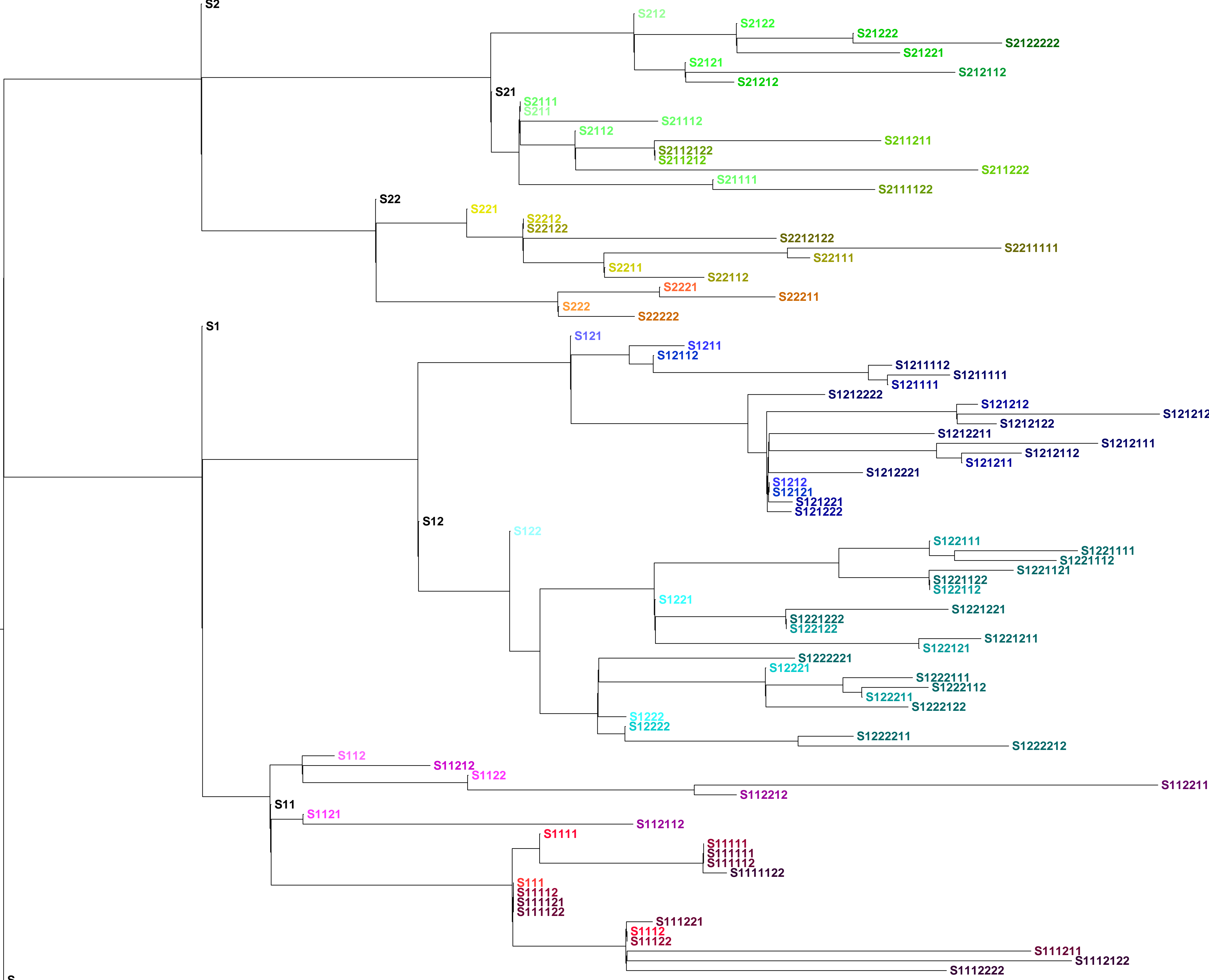

Supplement: Additional file 1: — a Tree inferred by CSI Phylogeny pruning set to 10, contigs from day 1 used as reference. PDF. b Tree inferred by CSI Phylogeny pruning set to 10, contigs from day 1 used as reference. Newick file. (ZIP 6 kb) [file 12864_2016_3407_MOESM1_ESM.zip › S1a.pdf]

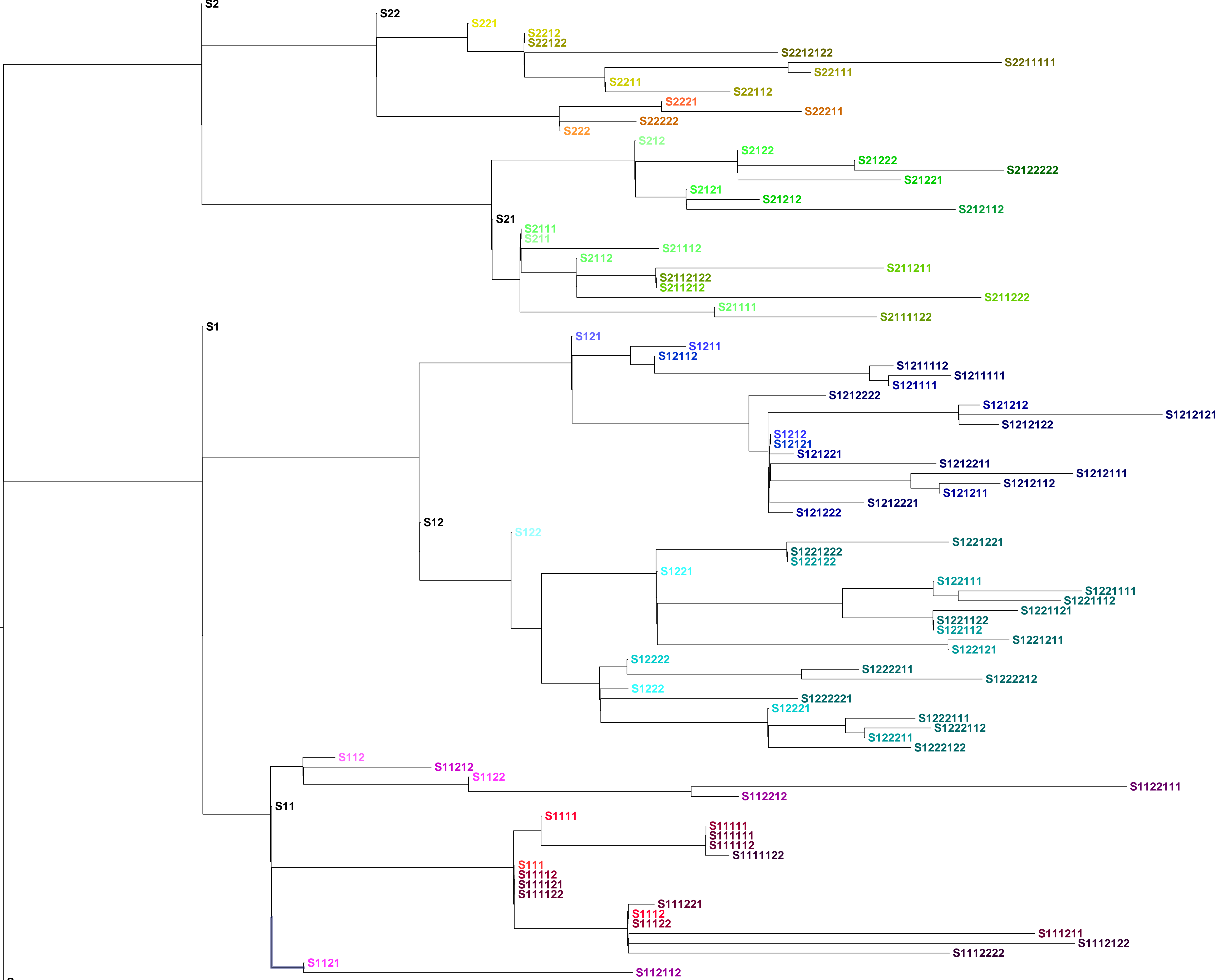

Supplement: Additional file 2: — a Tree inferred by CSI Phylogeny pruning disabled, E. coli MG1655 (NC_000913) used as reference. PDF. b Tree inferred by CSI Phylogeny pruning disabled, E. coli MG1655 (NC_000913) used as reference. Newick file. (ZIP 6 kb) [file 12864_2016_3407_MOESM2_ESM.zip › S2a.pdf]

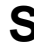

Supplement: Additional file 3: — a Tree inferred by NDtree, Z-score 1.96, Neighbor Joining tree, contigs from day 1 used as reference. PDF. b Tree inferred by NDtree, Z-score 1.96, Neighbor Joining tree, contigs from day 1 used as reference. Newick file. (ZIP 5 kb) [file 12864_2016_3407_MOESM3_ESM.zip › S3a.pdf]

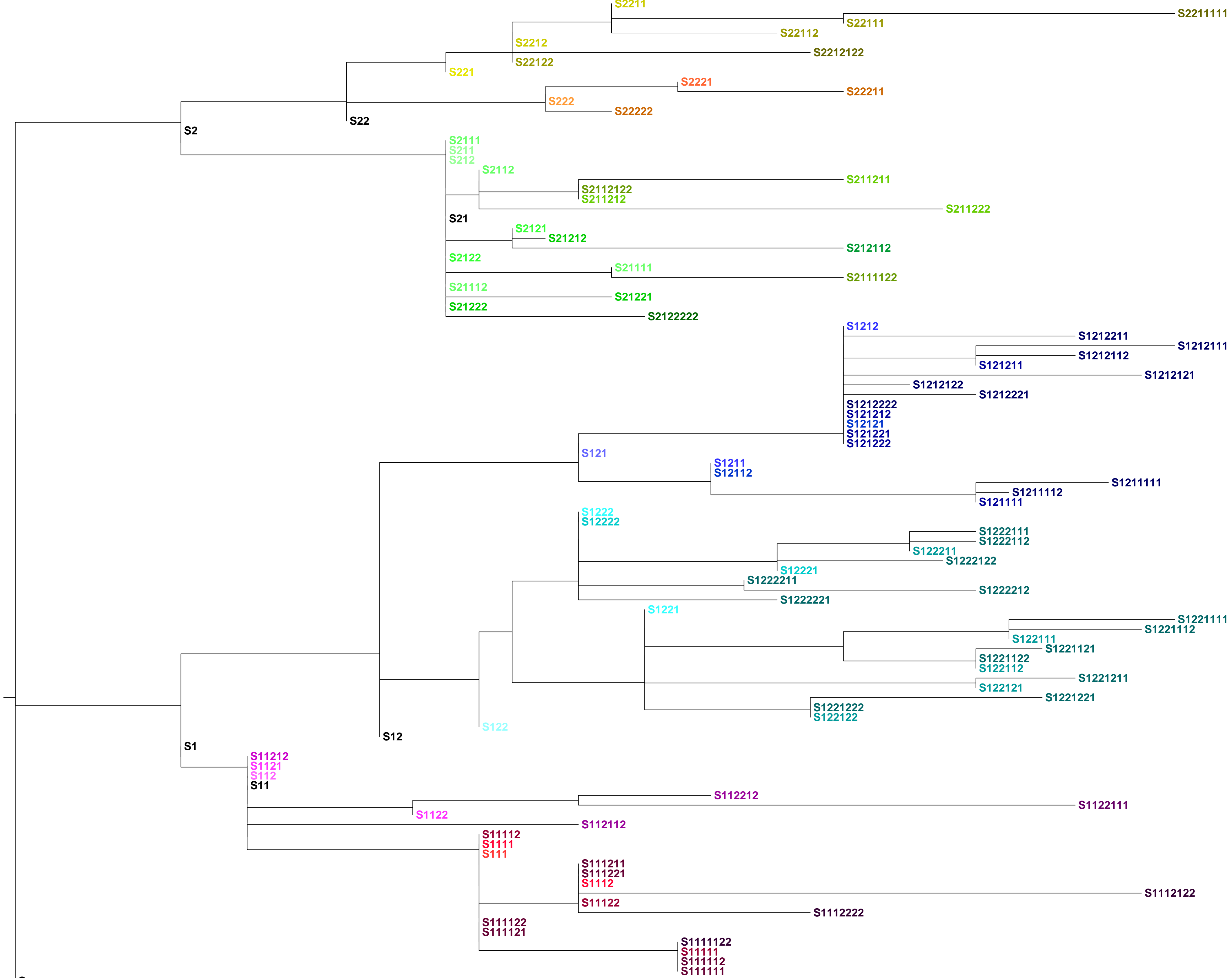

Supplement: Additional file 4: — a Tree inferred by NDtree, Z-score 1.64, Neighbor Joining tree, contigs from day 1 used as reference. PDF. b Tree inferred by NDtree, Z-score 1.64, Neighbor Joining tree, contigs from day 1 used as reference. Newick file. (ZIP 5 kb) [file 12864_2016_3407_MOESM4_ESM.zip › S4a.pdf]

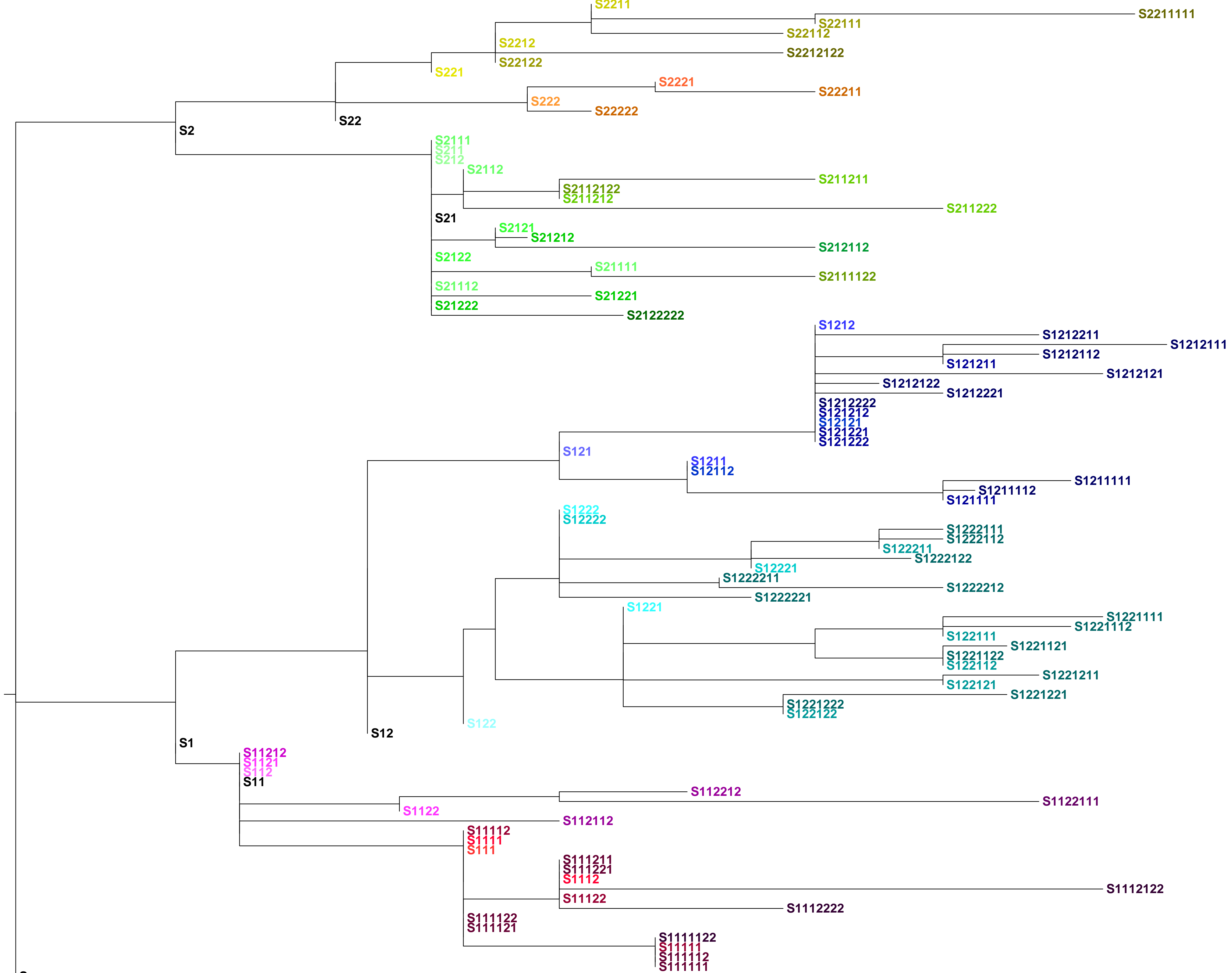

Supplement: Additional file 5: — a Tree inferred by NDtree, Z-score 1.96, Neighbor Joining tree, E. coli MG1655 (NC_000913) used as reference. PDF. b Tree inferred by NDtree, Z-score 1.96, Neighbor Joining tree, E. coli MG1655 (NC_000913) used as reference. Newick file. (ZIP 5 kb) [file 12864_2016_3407_MOESM5_ESM.zip › S5a.pdf]

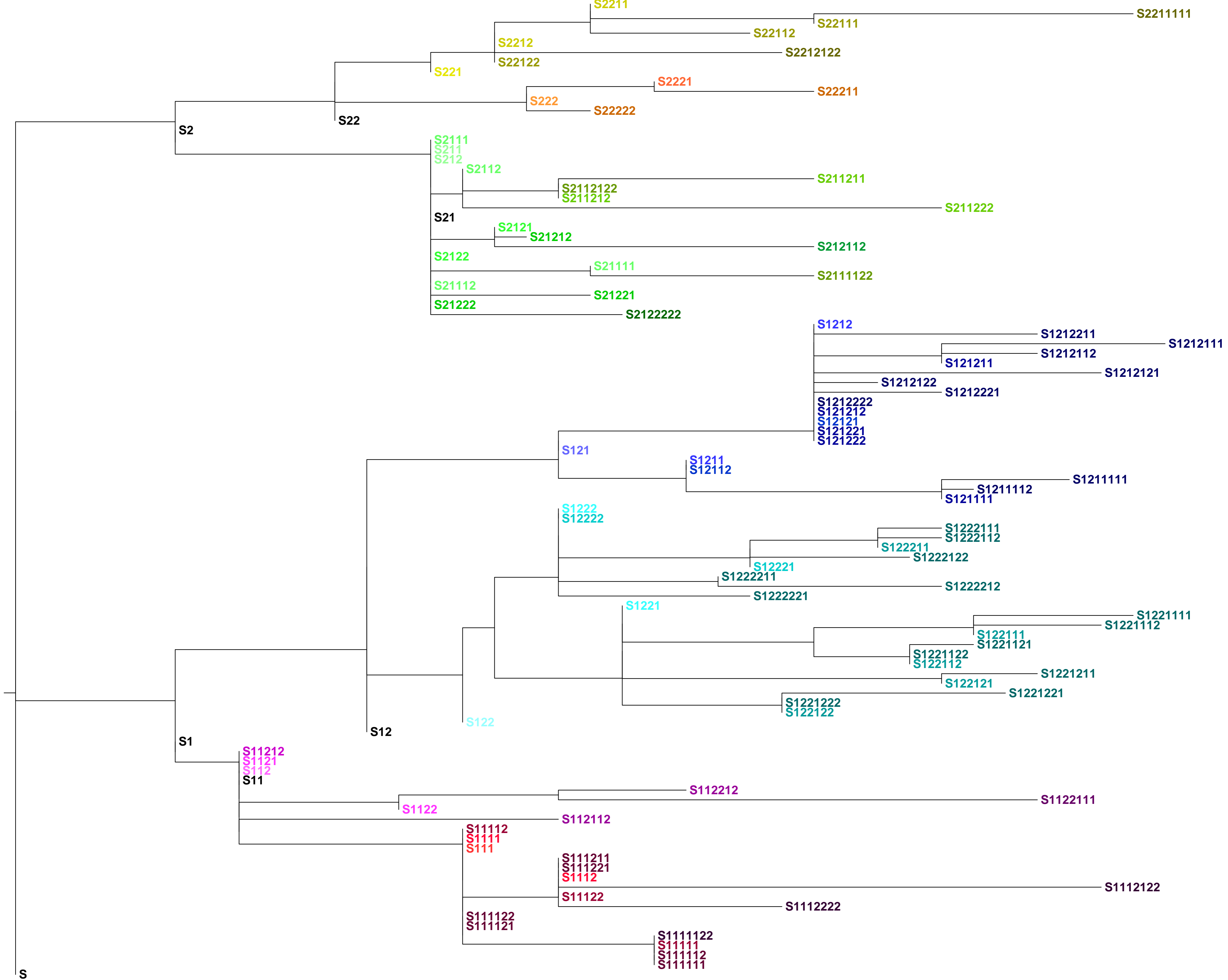

Supplement: Additional file 6: — a Tree inferred by NDtree, Z-score 1.96, Neighbor Joining tree, E. coli NC_012759 used as reference. PDF. b Tree inferred by NDtree, Z-score 1.96, Neighbor Joining tree, E. coli NC_012759 used as reference. Newick file. (ZIP 5 kb) [file 12864_2016_3407_MOESM6_ESM.zip › S6a.pdf]

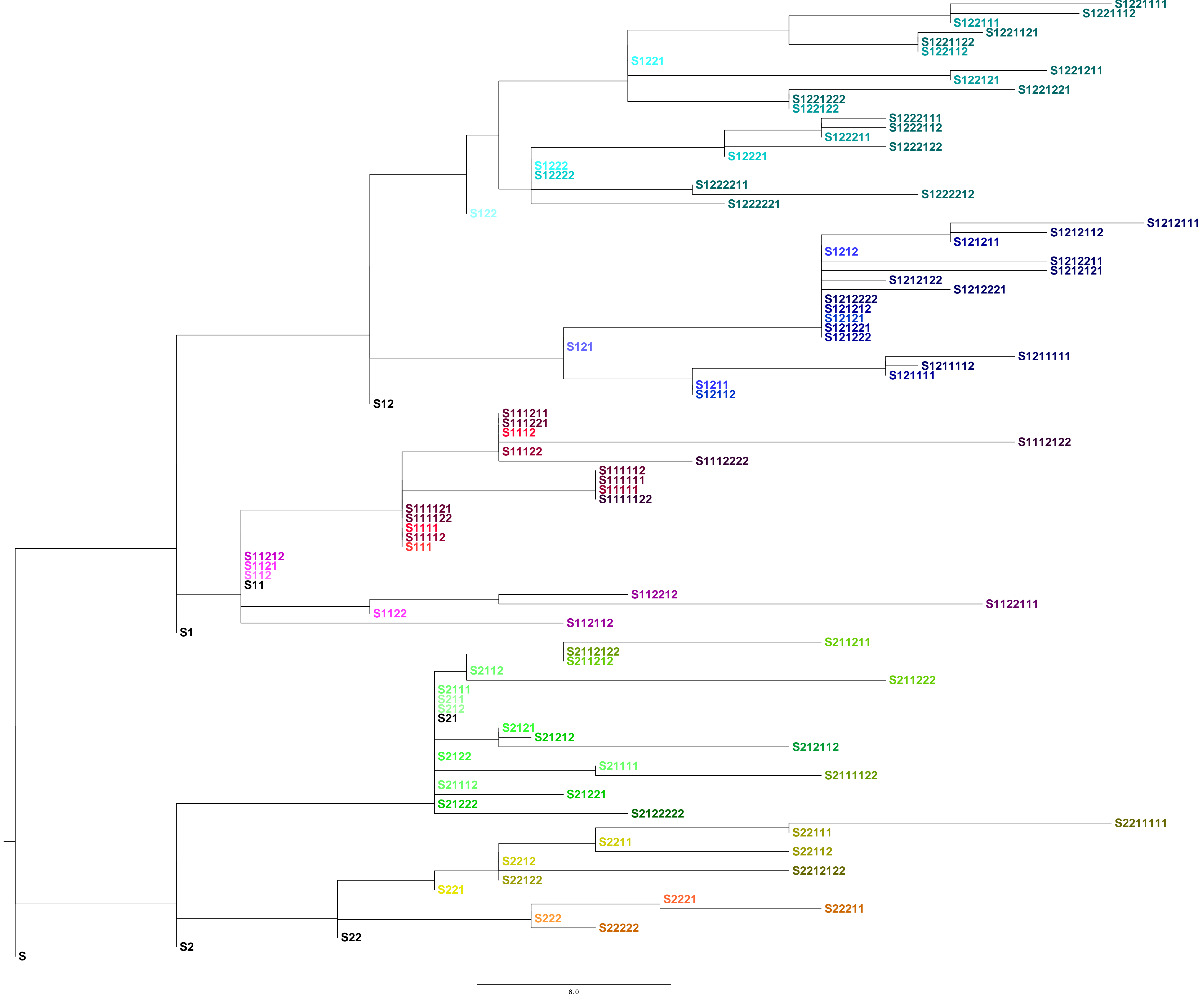

Supplement: Additional file 7: — a Tree inferred by NDtree, Z-score 1.96, Neighbor Joining tree, E. coli NC_017641 used as reference. PDF. b Tree inferred by NDtree, Z-score 1.96, Neighbor Joining tree, E. coli NC_017641 used as reference. Newick file. (ZIP 5 kb) [file 12864_2016_3407_MOESM7_ESM.zip › S7a.pdf]

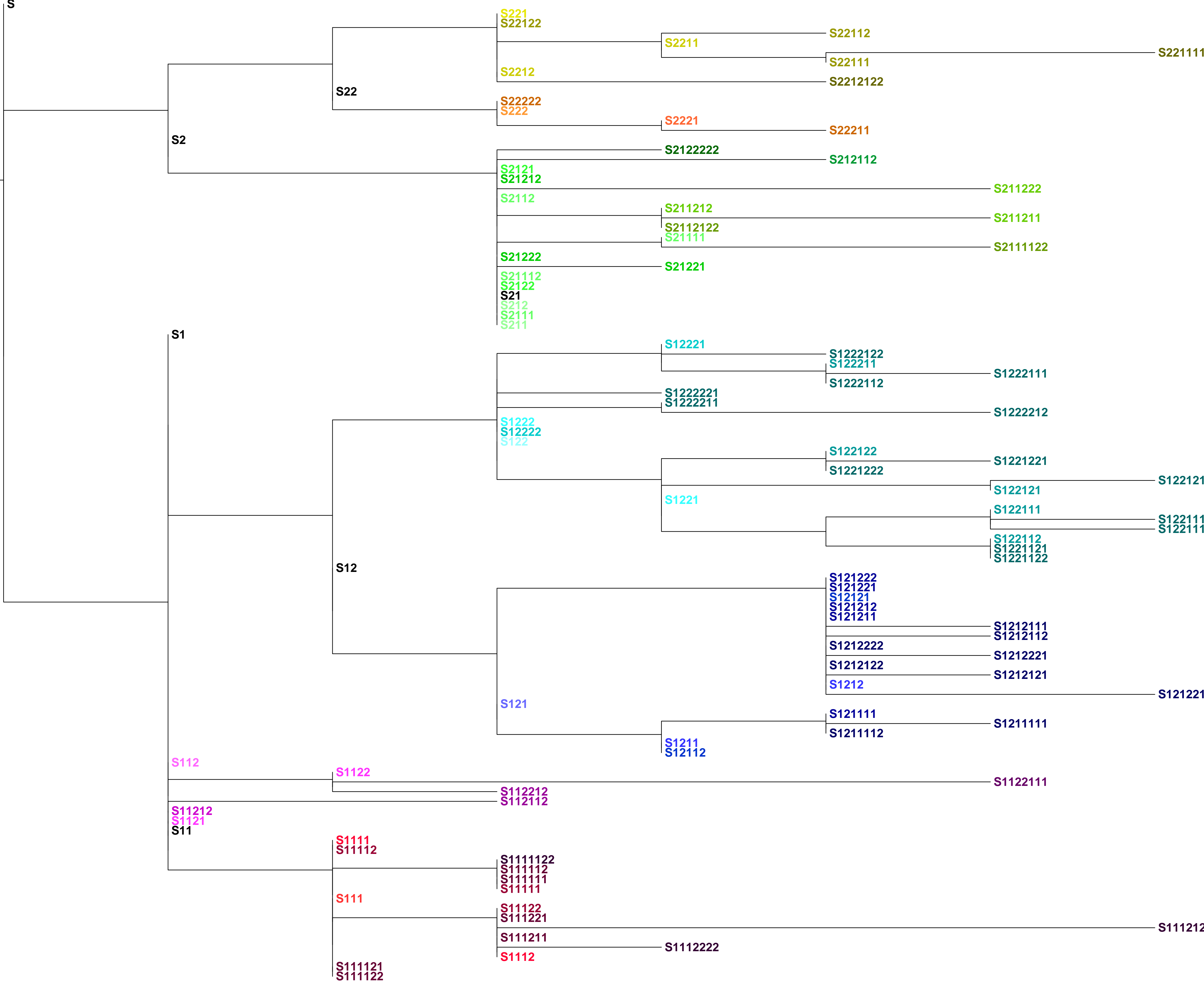

Supplement: Additional file 8: — a Tree inferred by REALPHY, PhyML, NC_000913, NC_012759 and NC_017641 used as reference. PDF. b Tree inferred by REALPHY, PhyML, NC_000913, NC_012759 and NC_017641 used as reference. Newick file. (ZIP 6 kb) [file 12864_2016_3407_MOESM8_ESM.zip › S8a.pdf]

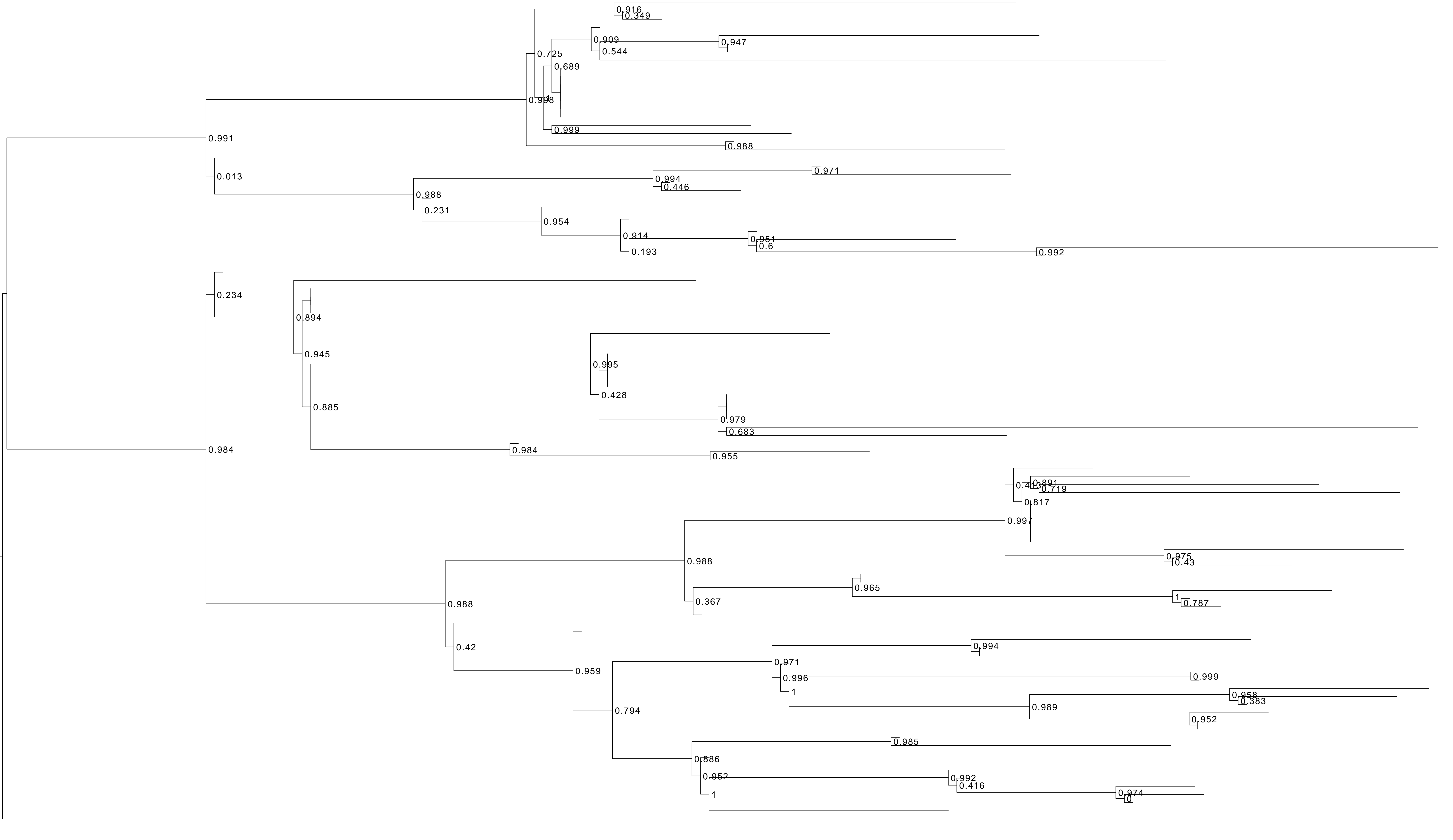

Supplement: Additional file 11: — Tree inferred by FastTree on the alignment from the pairwise NDtree analysis. Bootstrap values shown. No isolate names. (PDF 4 kb) [file 12864_2016_3407_MOESM11_ESM.pdf]

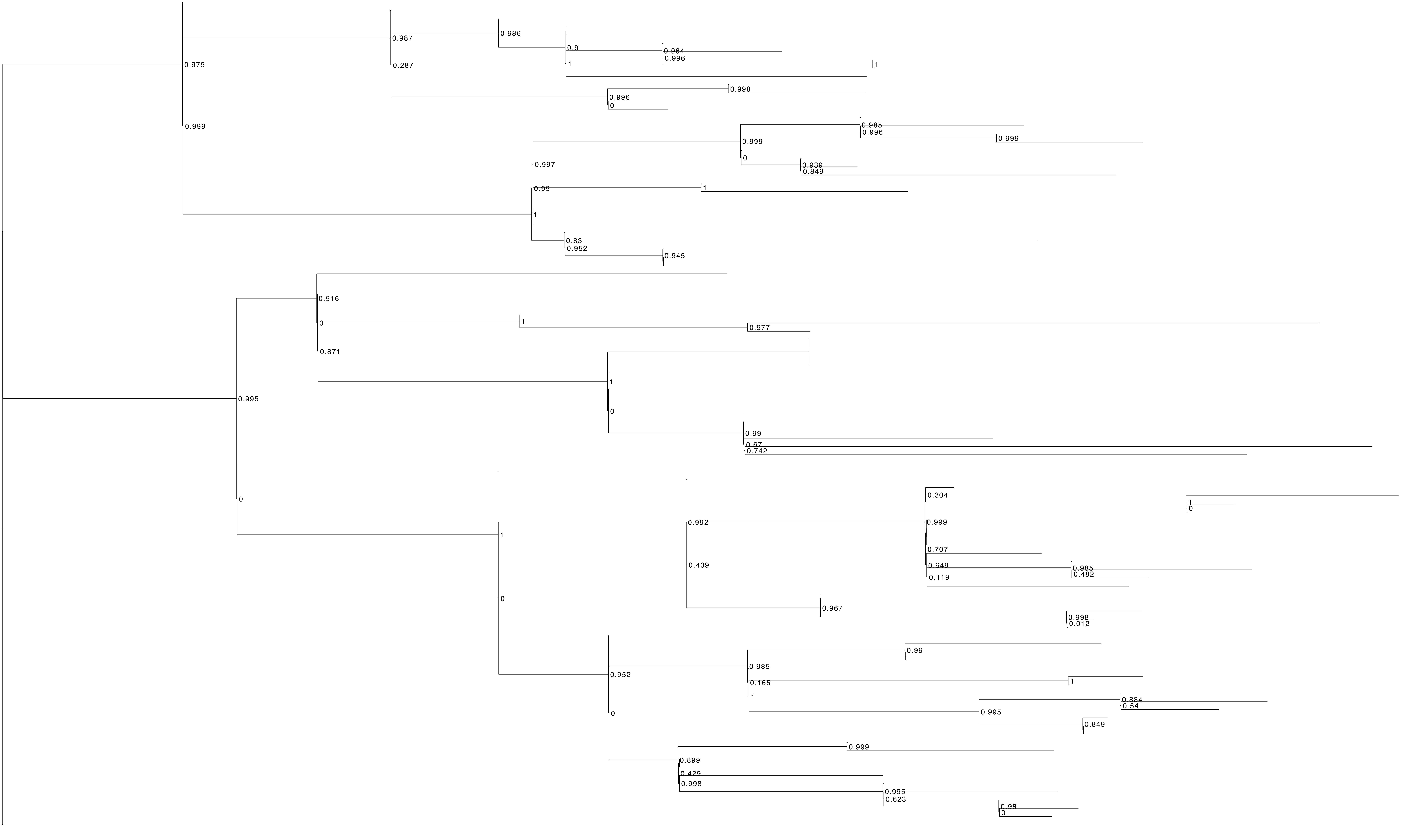

Supplement: Additional file 12: — Tree inferred by CSI Phylogeny, same tree as Fig. 3. Bootstrap values shown. No isolate names. (PDF 20 kb) [file 12864_2016_3407_MOESM12_ESM.pdf]

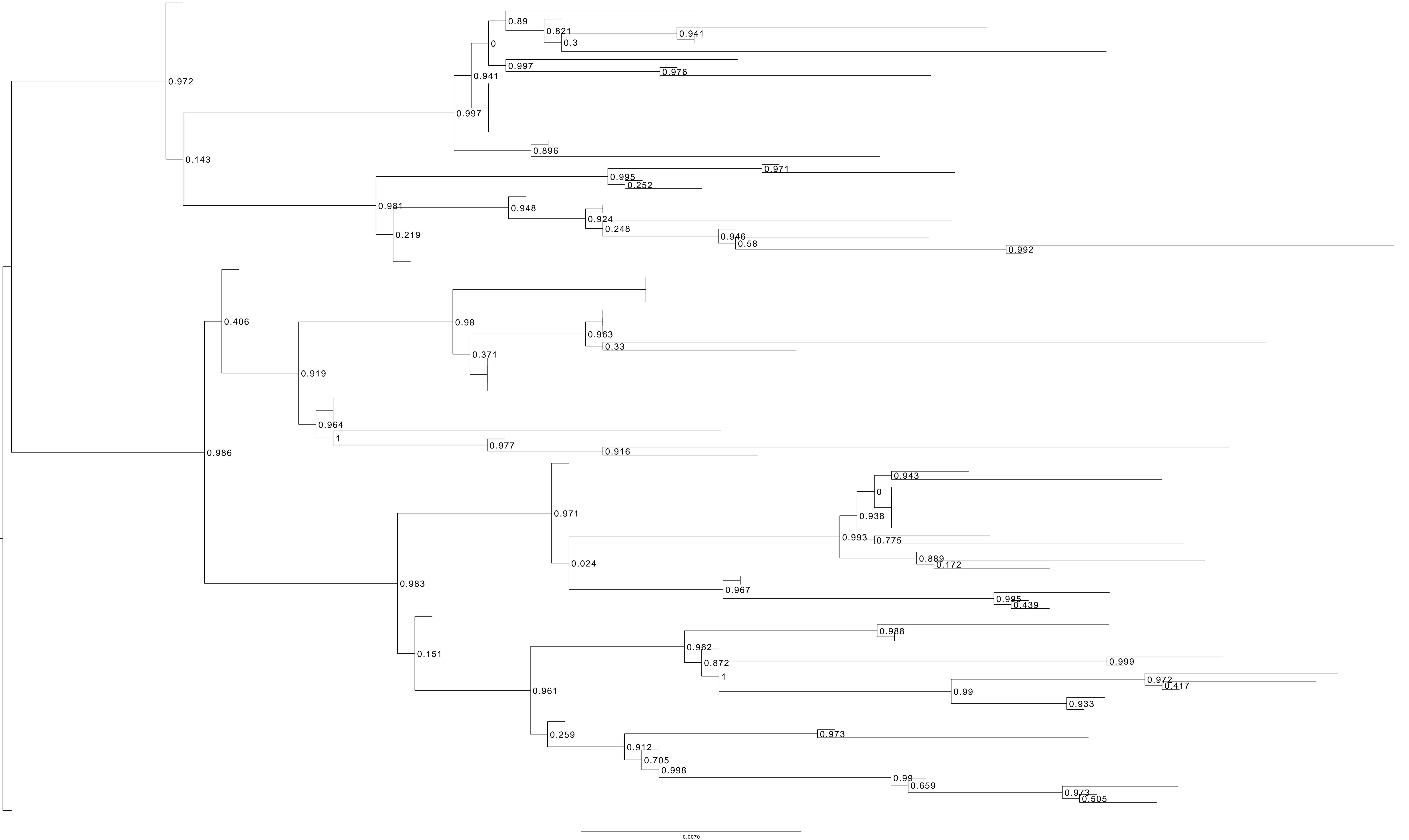

Supplement: Additional file 13: — Tree inferred by FastTree on the alignment from the REALPHY phyML analysis with 2 reference genomes. Bootstrap values shown. No strain names. (PDF 4 kb) [file 12864_2016_3407_MOESM13_ESM.pdf]
