# Supplementary material for: Bacterial whole genome-based phylogeny: construction of a new benchmarking dataset and assessment of some existing methods
Source: BMC Genomics. 2017 Jan 5;18:19. doi: 10.1186/s12864-016-3407-6 (PMC5217230; doi:10.1186/s12864-016-3407-6)
Supplement: Additional file 10: — a Tree inferred by REALPHY, RAxML, NC_000913 and NC_012759 used as reference. PDF. b Tree inferred by REALPHY, RAxML, NC_000913 and NC_012759 used as reference. Newick file. (ZIP 6 kb) [file S10a.pdf › S10a.pdf]

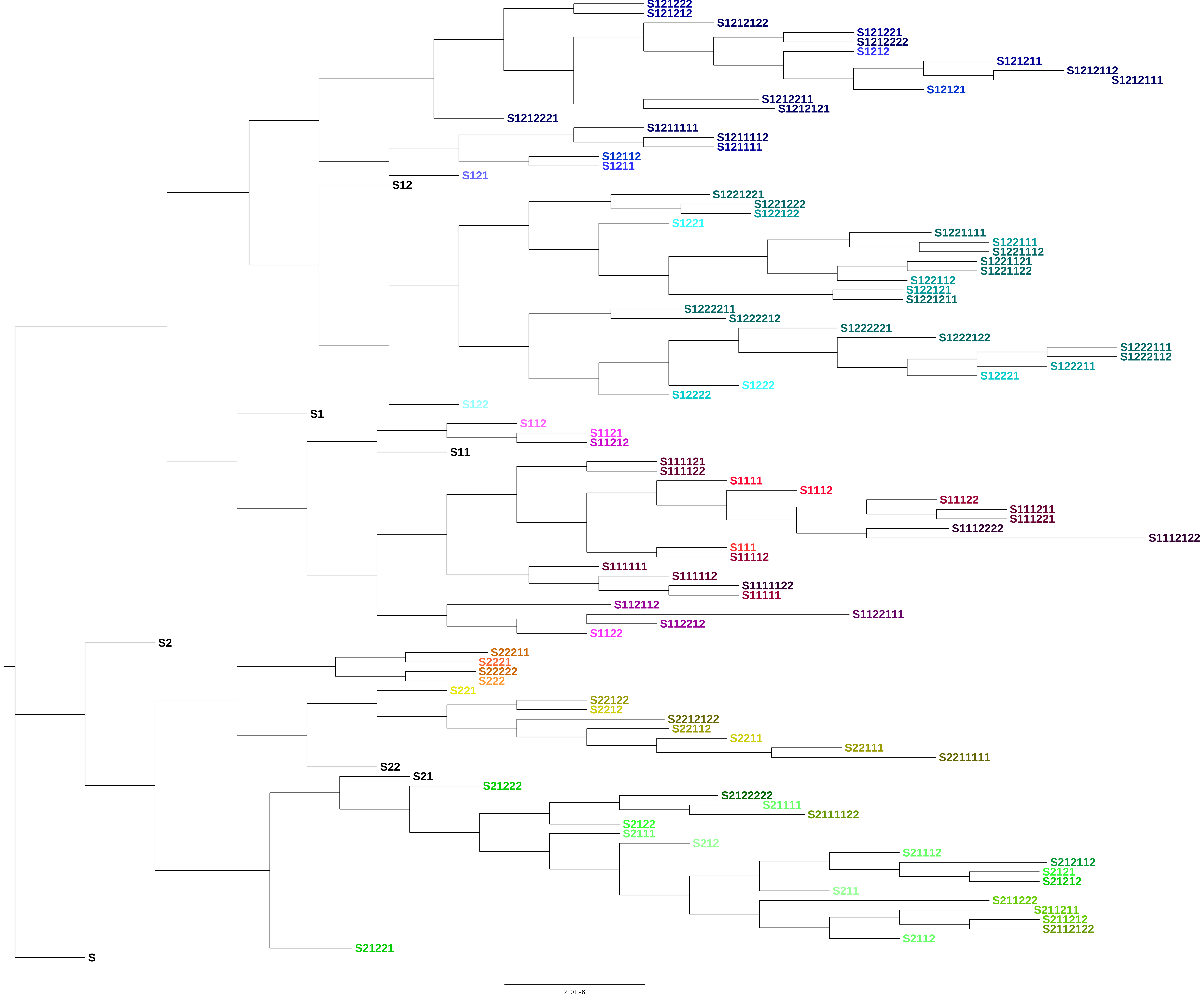
S121222
S121212
S1212122
S121221
S1212222
S1212
S121211
S1212112
S1212111
S12121
S1212211
S1212121
S1212221
S1211111
S1211112
S121111
S12112
S1211
S121
S12
S1221221
S1221222
S122122
S1221
S1221111
S122111
S1221112
S1221121
S1221122
S122112
S122121
S1221211
S1222211
S1222212
S1222221
S1222122
S1222111
S1222112
S122211
S12221
S1222
S12222
S122
S1
S112
S1121
S11212
S11
S111121
S111122
S1111
S1112
S11122
S111211
S111221
S1112222
S1112122
S111
S11112
S111111
S111112
S1111122
S11111
S112112
S1122111
S112212
S1122
S2
S22211
S2221
S22222
S222
S221
S22122
S2212
S2212122
S22112
S2211
S22111
S2211111
S22
S21
S21222
S2122222
S21111
S2111122
S2122
S2111
S212
S21112
S212112
S2121
S21212
S211
S211222
S211211
S211212
S2112122
S2112
S21221
S
2.0E-6
